# Epithelial Inflammatory iNOS–Nitrate Axis Enhances *P. gingivalis* Pathogenicity via *V. parvula* in Diabetes-Associated Periodontitis

**DOI:** 10.3390/ijms27177934

**Published:** 2026-09-06

**Authors:** Linhesheng Wei, Hui Liu, Zongshan Shen, Zhi Song, Shuheng Huang, Zhengmei Lin, Xin Huang

**Affiliations:** Guangdong Provincial Key Laboratory of Stomatology, Guanghua School of Stomatology, Hospital of Stomatology, Sun Yat-sen University, Guangzhou 510055, China; weilhsh@mail2.sysu.edu.cn (L.W.); liuh237@mail2.sysu.edu.cn (H.L.);

**Keywords:** oral mucosal inflammation, iNOS, nitrate, *Porphyromonas gingivalis*, *Veillonella parvula*, host–microbe interaction, diabetes-associated periodontitis

## Abstract

The mechanisms by which host inflammatory responses reshape the pathogenic potential of oral microbiota in mucosal inflammatory diseases remain poorly defined. Metagenomic sequencing of subgingival plaque and single-cell RNA sequencing of gingival tissues were performed in patients with diabetes-associated periodontitis (DP) and non-diabetic periodontitis (P). A diabetic mouse model was used to evaluate inflammation and bone resorption following oral inoculation with *Porphyromonas gingivalis* (*P. gingivalis*), *Veillonella parvula* (*V. parvula*), or both. Human gingival epithelial cells (HGECs) were cultured under high glucose conditions with or without an iNOS inhibitor 1400 W to assess iNOS and nitrate levels. A *P. gingivalis*–*V. parvula* co-culture system with nitrate supplements was used to evaluate *P. gingivalis* growth and virulence. Higher abundance of *P. gingivalis* and *V. parvula* was found in DP subgingival plaque. In diabetic mice, these bacteria worsened bone resorption and increased iNOS^+^ epithelial cells. Single-cell sequencing showed higher iNOS expression in DP patients, linked to *V. parvula*. In vitro, high glucose increased iNOS and nitrate in HGECs, and these effects were reversed by 1400 W. With *V. parvula*, nitrate over 200 μM enhanced *P. gingivalis* growth and virulence. Diabetic epithelial iNOS-derived nitrate boosts *P. gingivalis* pathogenicity through *V. parvula*, worsening periodontal inflammatory bone damage.

## 1. Introduction

The oral mucosa serves as a crucial barrier against microbes, situated near a complex microbial ecosystem of over 700 bacterial species vital for oral health [1,2]. In a typical balanced relationship between the host and oral microbiota, the mucosal epithelia release immune defenses, while the microbiota inhibits pathogen growth and maintain homeostasis [3,4]. This interaction is mutual and interdependent, with the host shaping microbial niches and the microbes influencing the host’s immune system [5]. For decades, research on inflammation–microbiota interactions has centered on the hypothesis that microbial dysbiosis triggers mucosal inflammation and disease, positing that dysbiosis leads to excessive inflammation and tissue damage in chronic conditions [6,7]. Nevertheless, in severe inflammatory disorders such as periodontitis and inflammatory bowel disease (IBD), the local microbiota remains persistent. Traditional anti-inflammatory treatments are often ineffective, and inflammation exhibits high recurrence rates [8,9]. The concept of “host inflammation actively regulating microbial pathogenicity” represents an innovative and significant area of research [10]. It is crucial for disrupting the cycle of dysbiosis and inflammation, a pattern frequently observed in the progression of chronic diseases.

Diabetes mellitus has been identified as a significant risk factor for chronic diseases, which are associated with considerable morbidity and mortality [11]. Periodontitis, a major inflammatory disease of the oral mucosa, is initiated by dysbiosis of the oral microbiota and is linked to the development of various systemic diseases [12,13,14]. Extensive clinical evidence has demonstrated a bidirectional relationship between diabetes and periodontitis, wherein each condition independently acts as a risk factor for the other, thereby collectively exacerbating disease progression [15]. Diabetic periodontitis (DP) patients often exhibit a more pronounced local inflammatory response and an increased pathogenic potential of the oral microbiota [16]. This situation may establish a positive feedback loop of pro-inflammatory responses, leading to more severe destruction of periodontal tissues, reduced treatment efficacy, and significantly higher recurrence rates of disease [9,17]. However, the extent to which the inflammatory state of the diabetic host influences the pathogenic potential of the oral microbiota remains unclear.

Inducible nitric oxide synthase (iNOS, encoded by the *NOS2* gene) serves as a crucial mediator in inflammatory responses by catalyzing the conversion of L-arginine to nitric oxide (NO) [18]. Upon inflammatory stimulation, the upregulation of iNOS results in the substantial production of NO, which functions as a critical antimicrobial molecule in the host’s innate defense mechanisms [19]. Macrophages exhibiting high iNOS expression are commonly employed as markers of inflammation. However, recent research in the field of the intestinal mucosa has revealed that host-derived inflammatory iNOS may confer a selective growth advantage to commensal bacteria possessing nitrate-reducing capabilities, such as *Veillonella parvula* (*V. parvula*), thereby influencing microbiota homeostasis [20]. This underscores the potential dual role of iNOS.

Socransky’s landmark classification of subgingival microorganisms into different “complexes” based on their biological characteristics and clinical inflammatory associations has been established [21]. *Veillonella parvula*, recognized as a “bridge microorganism” in the purple complex, facilitates interspecies interactions and enhances the colonization and proliferation of periodontal pathogens, including *Porphyromonas gingivalis* (*P. gingivalis*), the keystone pathogen in the red complex [22,23]. *P. gingivalis* virulence factors, like gingipains and DDP7, can degrade insulin-related proteins, possibly leading to insulin resistance and systemic metabolic issues [24,25]. However, in diabetic individuals, the interspecies interaction and virulence of key oral microorganisms remain poorly studied and understood.

Here, we used diabetes-associated periodontitis to investigate whether diabetes-associated alterations in the gingival epithelial microenvironment reshape microbial interactions and aggravate periodontal destruction. We identify a potential iNOS–nitrate axis in gingival epithelial cells that promotes *V. parvula*-dependent enhancement of *P. gingivalis* growth and virulence, providing a mechanistic link among the diabetic host environment, microbial cooperation, and periodontal tissue destruction.

## 2. Results

### 2.1. Elevated Abundance of P. gingivalis and V. parvula with Disrupted Homeostasis of Nitrate-Reducing Microbiota in the Subgingival Plaque of Diabetes-Associated Periodontitis Patients

**Figure 1:**
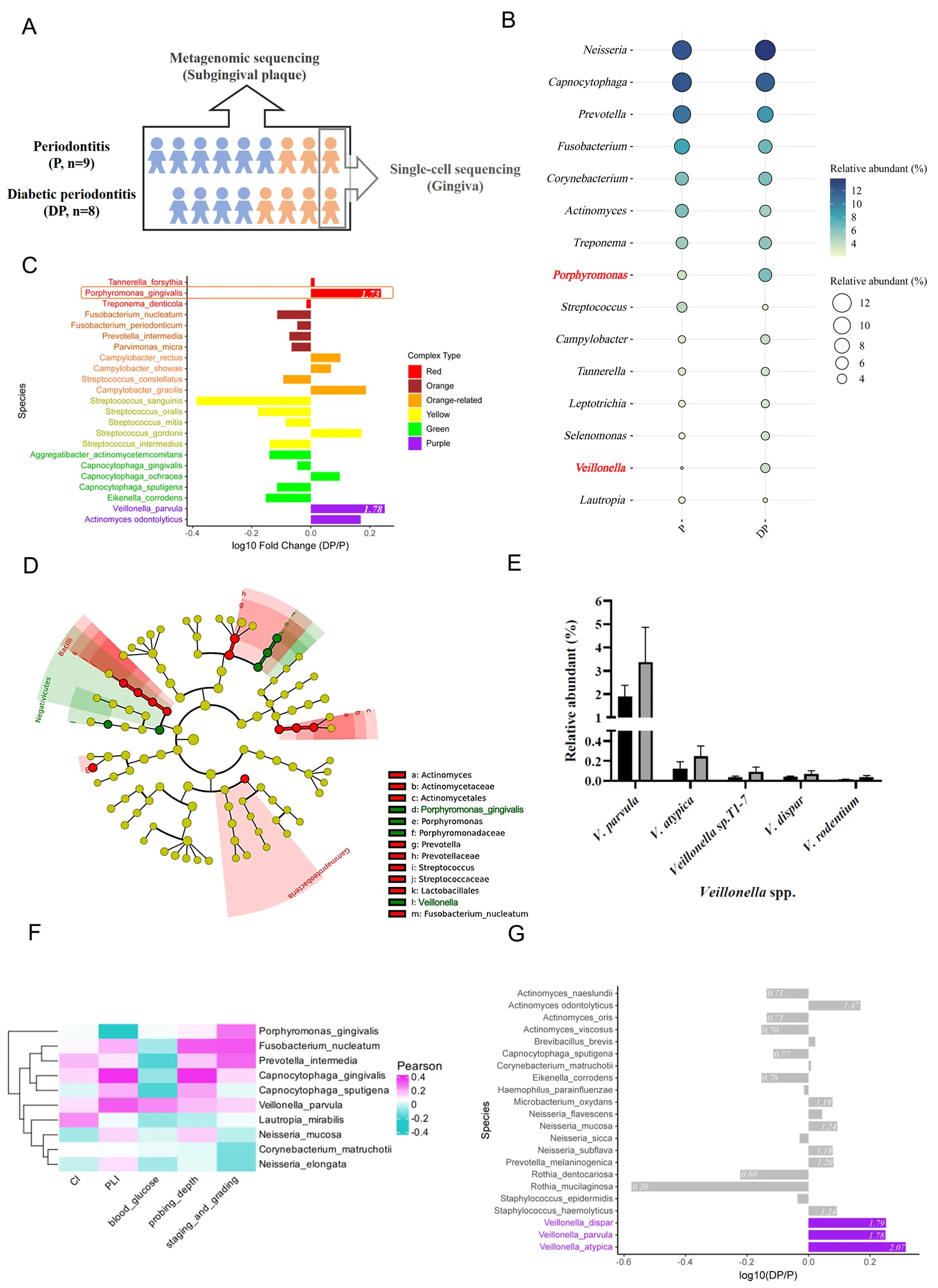
Elevated abundance of *P. gingivalis* and *V. parvula* with disrupted nitrate-reducing microbiota in the subgingival plaque of diabetes-associated periodontitis patients. (**A**) The schematic illustration provides an overview of the sample collection and sequencing process. Subgingival plaque samples were collected from participants in both the diabetes-associated periodontitis (DP) and periodontitis (P) groups for metagenomic sequencing, with sample sizes of *n* = 9 for the P group and *n* = 8 for the DP group. Gingival tissue samples were collected from one female participant from each group for single-cell RNA sequencing, amounting to *n* = 1 for each group. The orange figures in the image represent female participants, while the blue figures represent male participants. (**B**) The bubble plot displays the relative abundance of the top 15 genera identified within the DP and P groups. (**C**) A bar chart illustrates the microbial community composition, segmented by periodontal complexes. Compared to the P group, the DP group exhibited a 1.73-fold higher abundance of *P. gingivalis* and a 1.78-fold higher abundance in *V. parvula* within subgingival plaque samples. (**D**) The LEfSe analysis highlights biomarkers among the top 40 dominant species with a linear discriminant analysis (LDA) score of 4.0. *P. gingivalis* and the *Veillonella* genus were identified as key biomarkers for the DP group. (**E**) A bar chart depicting species abundance within the *Veillonella* genus reveals an upward trend in all *Veillonella* species in the DP group, with *V. parvula* showing a distinct presence in plaque enrichment. (**F**) The heat map of Pearson correlations delineates the relationships among microbiota and clinical indices, indicating positive correlations between periodontal pathogens like *P. gingivalis* and *T. forsythia* with enhanced periodontitis severity, while *V. parvula* demonstrated a positive correlation with elevated blood glucose levels. (**G**) The bar chart presents the changes in the abundance of oral nitrate-reducing microorganisms. Disease-associated *Veillonella* species displayed a consistent increase in abundance, contrasting with a decline in health-associated *Rothia* species, signaling potential dysbiosis of the oral nitrate-reducing microbiota.

Descriptive statistical analysis of clinical baseline characteristics showed no significant differences between the DP and P groups in terms of gender, age, calculus index, plaque index, deepest periodontal probing depth, bleeding on probing, or periodontitis staging and grading (Table 1, Figure 1A).

For the microbiota composition analysis, the genus-level results showed that, among the top 15 most abundant microorganisms in the DP group, the abundances of the *Porphyromonas* and *Veillonella* genera were increased (Figure 1B). The species-level results showed that the abundance of *P. gingivalis* was 1.73-fold higher in the DP group than in the P group, and the abundance of *V. parvula* was 1.78-fold higher (Figure 1C). Linear discriminant analysis effect size (LEfSe) analysis identified *P. gingivalis* and the *Veillonella* genus as biomarkers for the DP group (Figure 1D, LDA = 4). All detected species within the *Veillonella* genus showed a consistent upward trend in abundance in the DP group, with *V. parvula* having the highest average abundance (Figure 1E). This consistent pattern across multiple *Veillonella* species suggests that the elevation of *V. parvula* may reflect conserved features of the genus.

Correlation analysis between clinical indicators and microbiota showed that, among the top 10 most abundant microorganisms, red complex members *P. gingivalis* and *Tannerella forsythia* were positively correlated with periodontitis staging and grading, while *V. parvula* was the only species significantly positively correlated with the blood glucose levels of enrolled patients (Figure 1F). Further analysis of the composition of oral nitrate-reducing microbiota revealed that the abundance of health-associated nitrate-reducing bacteria such as *Rothia* spp. was decreased in the DP group, while the abundances of disease-associated nitrate-reducing genera such as *Prevotella* spp. and *Veillonella* spp. were increased, indicating a potential dysbiosis in nitrate-reducing microbiota among DP individuals (Figure 1G).

### 2.2. Enhanced Periodontal Destruction Induced by P. gingivalis–V. parvula Co-Inoculation Is Dependent on the Diabetic Host State

Based on the characteristics of significant co-enrichment of *P. gingivalis* and *V. parvula* and disrupted homeostasis of nitrate-reducing microbiota in the subgingival plaque of DP patients, we further verified the periodontal pathogenicity of the two bacteria in in vivo animal experiments.

**Figure 2:**
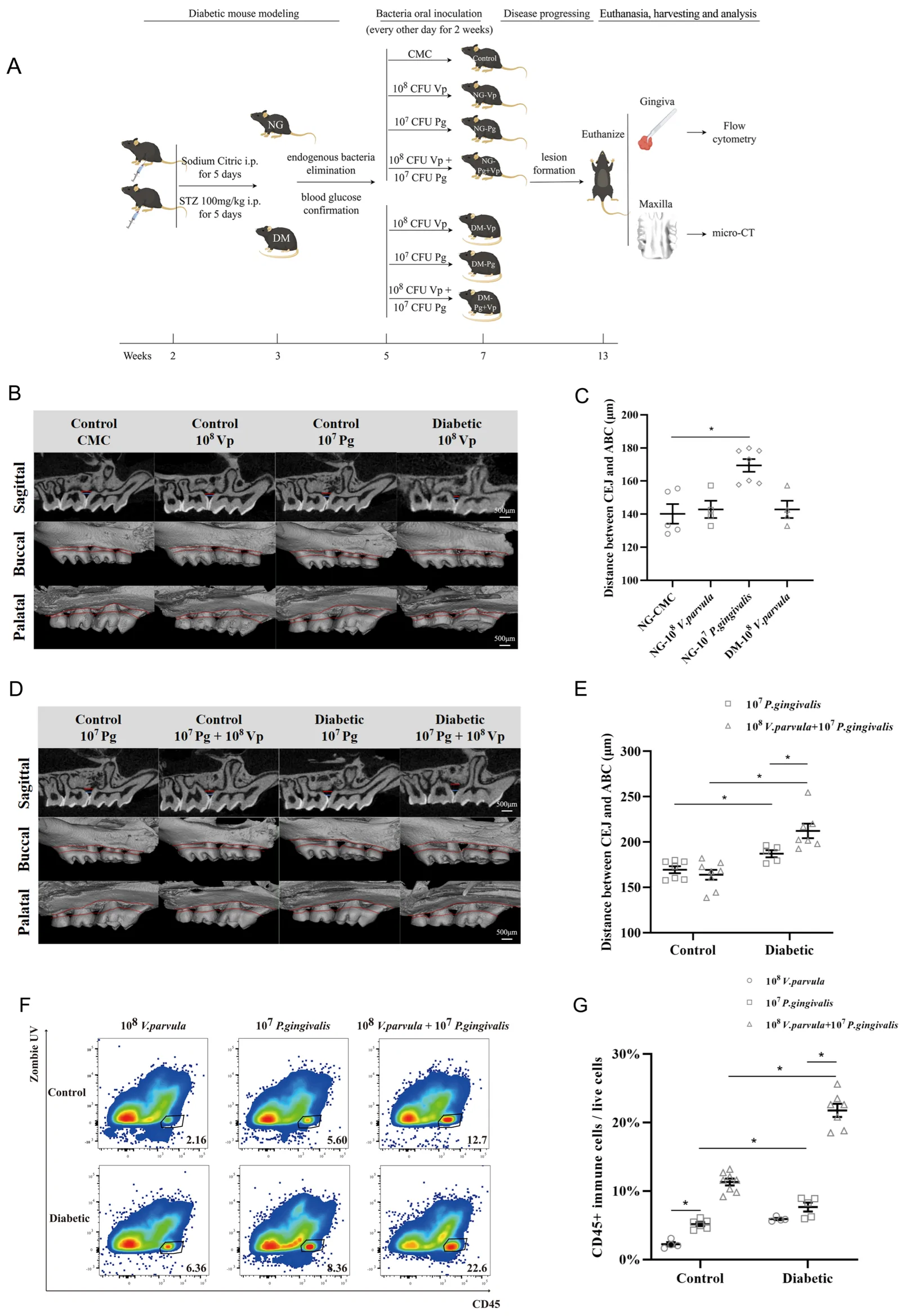
Enhanced inflammation and alveolar bone resorption by oral co-inoculation of *P. gingivalis* and *V. parvula* in diabetic mice. (**A**) Animal grouping and flowchart (generated using Figdraw). (**B**–**E**) Micro-CT reconstruction (**B**,**D**) and quantitative analysis (**C**,**E**) of interproximal alveolar bone resorption between the maxillary first and second molars (indicated by height reduction between red lines and blue lines, * *p* < 0.05). Oral inoculation with *P. gingivalis* induced alveolar bone resorption, whereas *V. parvula* inoculation failed to elicit detectable bone loss. Co-inoculation with *P. gingivalis* and *V. parvula* induced enhanced alveolar bone resorption, which was observed exclusively in diabetic mice. (**F**,**G**) Flow cytometry gating results (**A**) and quantitative analysis (**B**) for the CD45+ immune cell proportion among total live cells (* *p* < 0.05). Oral co-inoculation with *P. gingivalis* and *V. parvula* induced more pronounced alveolar bone resorption, with exacerbated severity observed in diabetic mice.

Single-inoculation results showed that inoculation with 10^7^ CFU of *P. gingivalis* alone induced significant alveolar bone resorption in both healthy and diabetic mice, while inoculation with 10^8^ CFU of *V. parvula* alone did not cause detectable alveolar bone loss in either group (Figure 2B,C, *p* < 0.05), confirming that *V. parvula* itself has no significant periodontal pathogenicity.

Dual-bacteria co-inoculation results showed that, in diabetic mice, the alveolar bone resorption in the *V. parvula* and *P. gingivalis* co-inoculation group was 1.14-fold higher than that in the *P. gingivalis* single-inoculation group. In contrast, in healthy mice, there was no significant statistical difference in alveolar bone resorption volume between the co-inoculation group and the *P. gingivalis* single-inoculation group, with a decreasing trend observed (Figure 2D,E, *p* < 0.05). The two-way ANOVA results showed a significant interaction between bacterial inoculation type and diabetic status in regulating alveolar bone resorption (Supplementary Table S3, *p* < 0.05), confirming that the enhanced bone-destructive effect of *P. gingivalis* and *V. parvula* is dependent on the diabetic host state.

Detection of gingival inflammation levels showed that, in both healthy and diabetic mice, the proportion of CD45^+^ immune cells in gingival tissues was significantly higher in the *V. parvula* and *P. gingivalis* co-inoculation group than in the *P. gingivalis* single-inoculation group (Figure 2F,G, *p* < 0.05). Two-way ANOVA also confirmed a significant interaction between bacterial inoculation type and diabetic status in regulating gingival inflammation (Supplementary Table S4, *p* < 0.05). These results collectively confirm that *V. parvula* itself has no significant periodontal pathogenicity but can act as a key auxiliary bacterium to significantly enhance the pro-inflammatory and bone-destructive potential of *P. gingivalis* only in the diabetic host microenvironment.

### 2.3. Upregulated Inflammatory iNOS Expression in Human Gingival Epithelial Cells Correlates Positively with V. parvula Abundance in the Subgingival Microbiota

**Figure 3:**
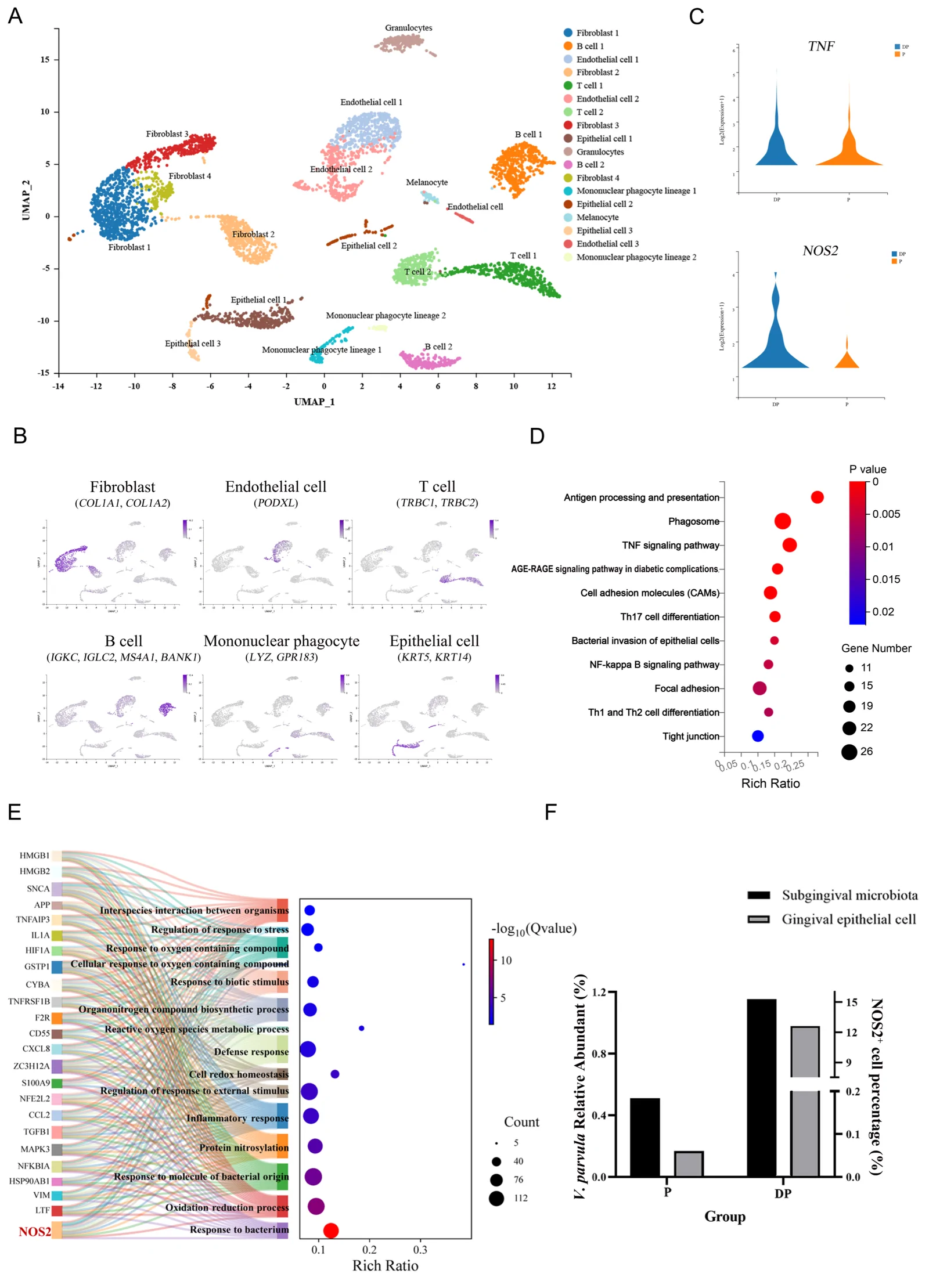
Upregulated inflammatory iNOS expression in human gingival epithelial cell correlates positively with *V. parvula* abundance in subgingival microbiota. (**A**,**C**) UMAP plot of and marker genes for cell clusters in the DP and P group. (**B**) Violin map of inflammatory response-related gene (*TNF*, *NOS2*) expression in the P and DP groups. (**D**) Bubble plot shows KEGG enrichment analysis of differentially expressed genes in all cell clusters between the P and DP groups; functional pathways related to epithelium were significantly enriched. (**E**) The Sankey bubble diagram illustrates inflammation-related differentially expressed genes and pathway enrichment in the epithelial cell cluster. *NOS2* was broadly enriched across multiple pathways, including oxidative stress, reactive oxygen species metabolism, redox homeostasis, nitrosative stress, and interspecies interaction. (**F**) Bar chart of the relative abundance of *V. parvula* in subgingival microbiota and the proportion of *NOS2*^+^ epithelial cells in gingiva.

In order to investigate the impact of diabetes on the local microenvironment and to identify host factors potentially contributing to local microbial dysbiosis, we conducted single-cell sequencing of gingival samples obtained from patients with clinical periodontitis (P) and those with diabetes-associated periodontitis (DP). UMAP unsupervised clustering analyzed a total of 18,472 cells and identified 18 distinct cell subsets (Figure 3A,B). Differential gene expression analysis showed that the expression levels of the inflammatory cytokine *TNF* and the inflammation-related gene *NOS2* (encoding iNOS) in the gingival tissues of the DP group were upregulated compared with the P group (Figure 3C). KEGG pathway enrichment analysis of upregulated genes in the DP group showed significant enrichment in pathways related to epithelial barrier functions such as antigen processing, cell adhesion, bacterial invasion, and tight junctions, diabetes-related pathways such as AGE-RAGE signaling, and inflammation-related pathways such as TNF signaling (Figure 3D, *p* < 0.05), suggesting that diabetes-induced molecular changes in gingival tissues are mainly concentrated in epithelial inflammation and dysfunction.

Further focusing on epithelial cell subsets marked by *KRT5* and *KRT14*, enrichment analysis of inflammation-related genes showed significant enrichment in defense response, bacterial invasion, and host–microbe interspecies interaction pathways, as well as biological processes such as response to oxygen-containing compounds, cellular redox homeostasis, and protein nitrosylation (Figure 3E, *Q* < 0.05). Among these, *NOS2* had the broadest pathway coverage, showing significant enrichment in oxidative stress, reactive oxygen species metabolism, redox homeostasis, nitrosative stress, and interspecies interaction pathways (Figure 3E, *Q* < 0.05). Paired analysis of single-cell sequencing and metagenomic sequencing data showed that the proportion of *NOS2*^+^ epithelial cells in gingival tissues was positively correlated with the abundance of *V. parvula* in subgingival plaque (Figure 3F), suggesting its potential role in DP epithelial inflammatory responses and host–microbe interactions.

**Figure 4:**
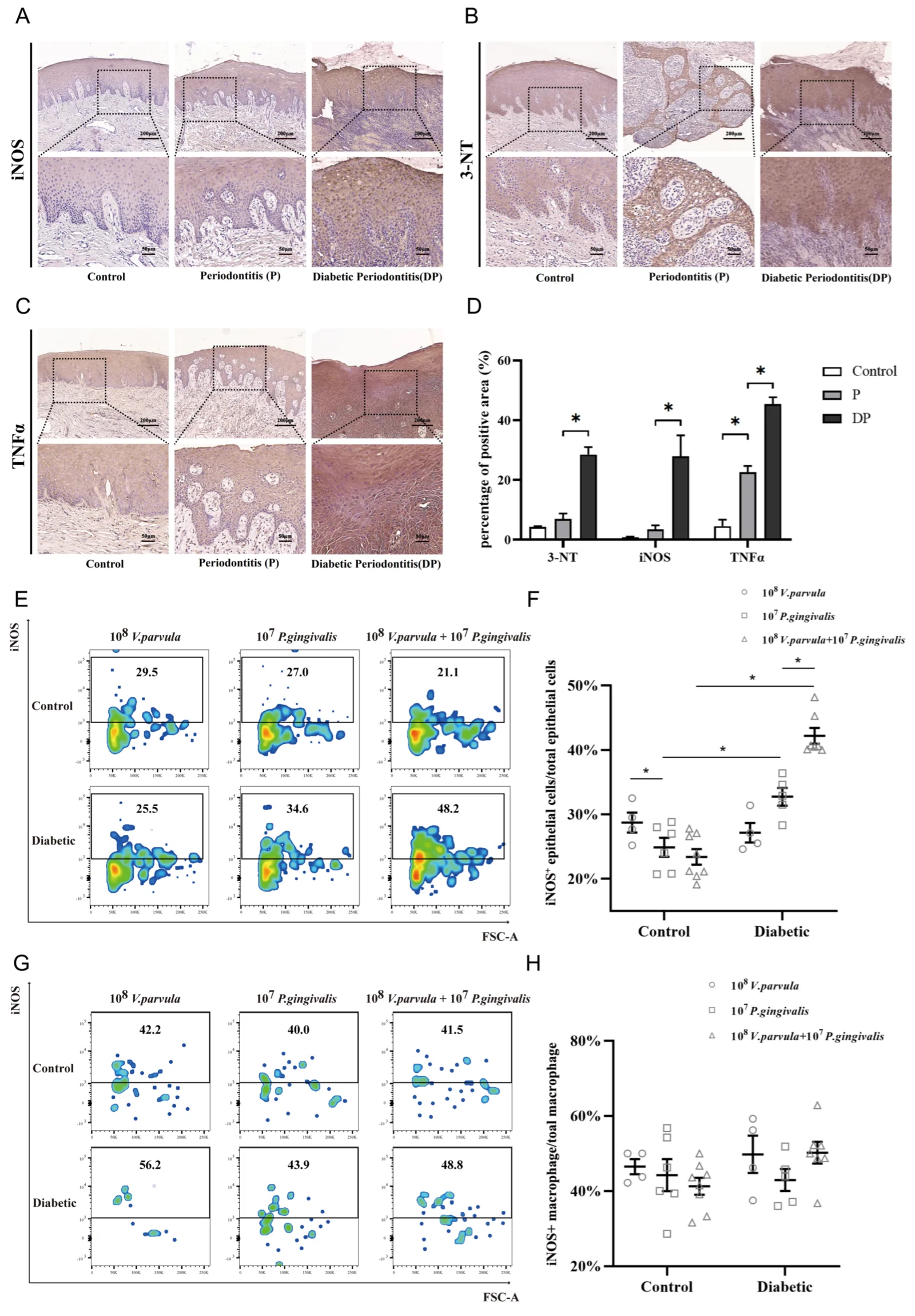
Increased iNOS^+^ gingival epithelial cells in diabetic mouse and human samples. (**A**–**D**) Representative samples and quantitative analysis of TNFα, iNOS, and 3-NT immunohistochemical staining in gingiva from the P and DP groups. Scale bars: 200 μm or 50 μm. Data are shown as the mean ± *s.e.m.* of three independent experiments (*n* = 3, * *p* < 0.05). (**E**,**F**) Flow cytometry gating results (**E**) and quantitative analysis (**F**) for iNOS^+^ epithelial cell proportion relative to total epithelial cells. (**G**,**H**) Flow cytometry gating results (**G**) and quantitative analysis (**H**) for iNOS^+^ epithelial macrophage proportion relative to total macrophages. (* *p* < 0.05). (**F**,**H**) Each value represents the mean ± *s.e.m.* of biological replicates (*n* = 4 in Control-10^8^ *V. parvula* group, *n* = 6 in Control-10^7^ *P. gingivalis* group, *n* = 8 in Control-10^8^ *V. parvula* + 10^7^ *P. gingivalis* group, *n* = 4 in Diabetic-10^8^ *V. parvula* group, *n* = 5 in Diabetic-10^7^ *P. gingivalis* group, *n* = 6 in Diabetic-10^8^ *V. parvula* + 10^7^ *P. gingivalis* group, * *p* < 0.05). Co-inoculation with *P. gingivalis* and *V. parvula* significantly increased the proportion of iNOS^+^ epithelial cells (not macrophages) in diabetic mice. The increase in iNOS^+^ epithelial cell proportion closely paralleled the severity of alveolar bone destruction in groups exhibiting bone loss.

We collected clinical human gingival tissues for further validation. The gingival tissues exhibited typical histological features of stratified squamous epithelium and rete pegs, and the increased infiltration of inflammatory cells indicated that both the P group and the DP group were in an inflammatory state. The immunohistochemical staining results for human gingival tissues were consistent with the single-cell sequencing results. TNFα expression was significantly upregulated in the P group compared with healthy controls (*p* < 0.05), while the expression levels of iNOS, TNFα, and the nitrosative stress marker 3-nitro-L-tyrosine (3-NT) in the DP group were increased by 8.1-fold, 2.0-fold, and 3.0-fold, respectively, compared with the P group, and their expression was mainly localized in epithelial cells (Figure 4A–D, *p* < 0.05). Negative control images were separately presented in Supplementary Materials (Figures S3–S6). The upregulation of TNFα in the DP group represents a higher inflammatory state, which is consistent with the single-cell sequencing result. 3-Nitrotyrosine (3-NT) serves as a biomarker indicative of the generation of nitric oxide-derived reactive nitrogen species (RNS) and the occurrence of nitrosative stress, while nitrate is a byproduct of nitrosative stress decomposition. Elevated concentrations of 3-NT may indicate increased nitrosative stress within epithelial tissues, potentially leading to the degradation into greater quantities of nitrates.

Flow cytometric analysis of mouse gingival tissues further showed that, after co-inoculation with *V. parvula* and *P. gingivalis* or single inoculation with *P. gingivalis*, the proportion of iNOS^+^ epithelial cells was significantly higher in diabetic mice than in non-diabetic mice, while there was no significant statistical difference in the proportion of iNOS^+^ macrophages among all groups (Figure 4E–G, *p* < 0.05). Two-way ANOVA showed a significant interaction between bacterial inoculation and diabetic status in regulating the proportion of iNOS^+^ epithelial cells (Supplementary Table S5, *p* < 0.05), and the increasing trend of iNOS^+^ epithelial cell proportion was consistent with the degree of alveolar bone destruction in mice. These results collectively confirm that gingival epithelial cells are the primary source of iNOS overexpression under diabetic conditions, and the expression level of epithelial iNOS is significantly positively correlated with subgingival *V. parvula* abundance and the degree of periodontal tissue destruction.

### 2.4. iNOS-Mediated High Nitrate Microenvironment in Gingival Epithelium in the Context of Hyperglycemia Promotes P. gingivalis Virulence and Proliferation via V. parvula

The above clinical and animal experimental results collectively suggest that the epithelial iNOS–nitrate axis may be the core molecular mechanism mediating the synergistic pathogenicity of *V. parvula* and *P. gingivalis* in the diabetic host microenvironment. To verify this hypothesis, we further conducted in vitro high glucose stimulation cell experiments and bacterial co-culture experiments to clarify the specific effects of the iNOS–nitrate axis on regulating the interaction between the two bacteria.

**Figure 5:**
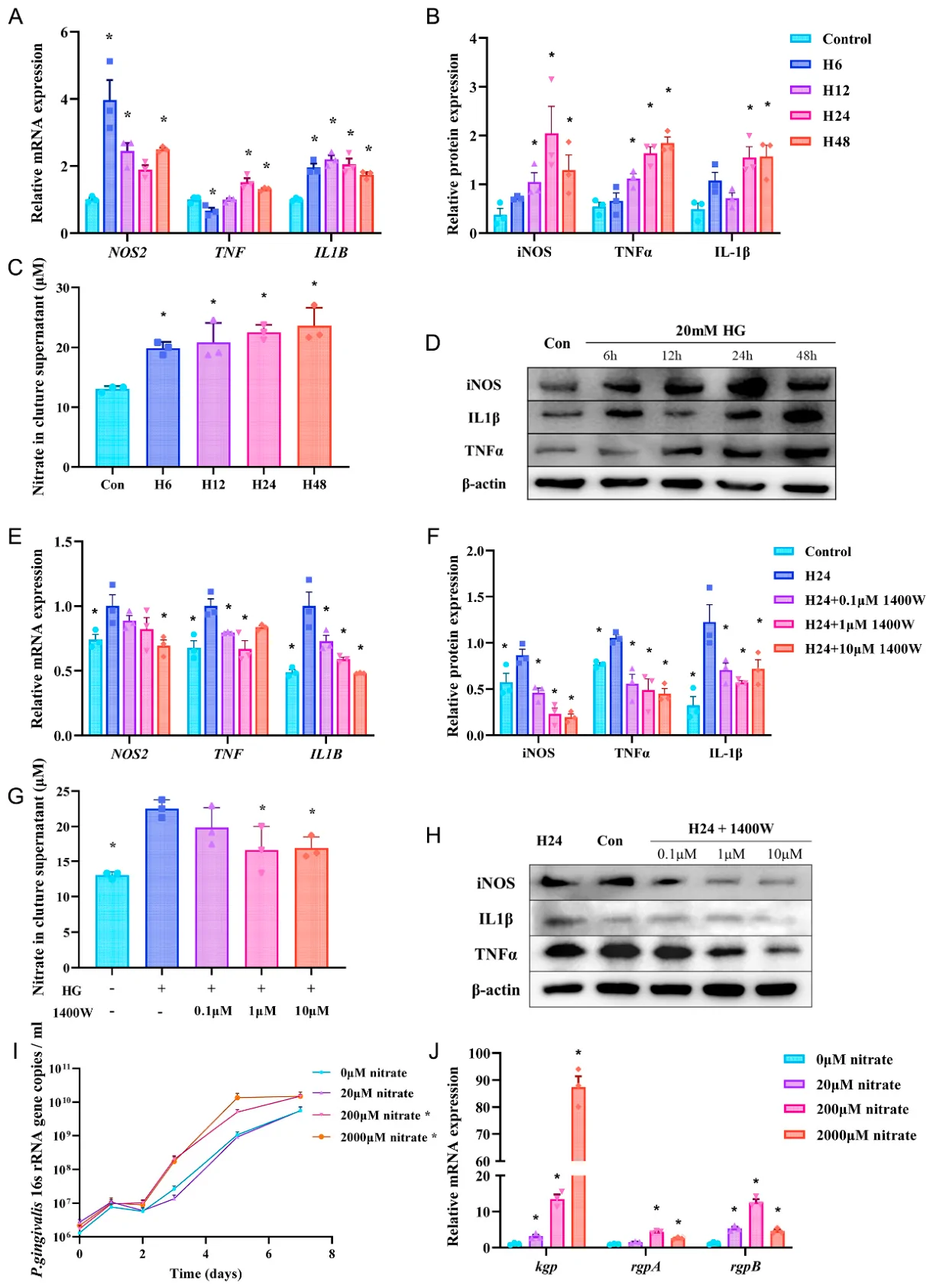
iNOS-mediated high nitrate microenvironment in gingival epithelium in the context of hyperglycemia promotes *P. gingivalis* virulence and proliferation via *V. parvula*. (**A**–**D**) High glucose stimulation (24 h and 48 h) induced significant upregulation of IL-1β, TNFα, and iNOS mRNA (**A**) and protein (**B**,**D**) expression levels in gingival epithelial cells, accompanied by increased nitrate concentrations in the culture supernatant (**C**) (* *p* < 0.05, compared with Control group). (**E**–**H**) The addition of 0.1 μM, 1 μM, and 10 μM 1400 W reversed the high glucose (24 h stimulation)-induced upregulation of IL-1β, TNFα, and iNOS mRNA (**E**) and protein (**F**,**H**) levels in gingival epithelial cells. The elevated nitrate concentration in the culture supernatant caused by high glucose stimulation was partially reversed (**G**) (* *p* < 0.05, compared with the H24 group). (**I**) Growth curve of *P. gingivalis* in a *P. gingivalis–V. parvula* dual species co-culture model supplemented with graded nitrate concentrations. Exogenous nitrate > 200 μM significantly enhanced *P. gingivalis* growth in *P. gingivalis–V. parvula* dual species co-cultures (groups exhibiting significantly higher biomass than 0 μM controls on any day after day 3 were considered significant). (**J**) Nitrate supplementation significantly upregulated *kgp*, *rgpA*, and *rgpB* expression in *P. gingivalis* (*p* < 0.05). All the western blotting and qRT-PCR experiments were repeated three independent times (*n* = 3), * *p* < 0.05. The data are shown as the mean ± *s.e.m*.

In the HGEC high glucose stimulation model, 20 mM high glucose stimulation upregulated the mRNA and protein expression of IL-1β and TNFα in HGECs in a time-dependent manner, with mRNA expression peaking at 12–24 h and protein expression peaking at 24–48 h (Figure 5A,B,D, *p* < 0.05). *NOS2* mRNA expression was significantly increased as early as 6 h after high glucose stimulation and peaked at 24 h, accompanied by a time-dependent increase in nitrate concentration in the culture supernatant (Figure 5A,C, *p* < 0.05).

Intervention experiments with gradient concentrations of the iNOS selective inhibitor 1400 W (0.1 μM, 1.0 μM, 10 μM) showed that 1400 W partially reversed the high glucose-induced upregulation of TNFα, IL-1β, and iNOS expression in a concentration-dependent manner and significantly reduced the elevated nitrate concentration in the 24 h culture supernatant (Figure 5E–H, *p* < 0.05).

Bacterial culture results showed that, in the *P. gingivalis* single culture system, exogenous nitrate supplementation had no significant effect on the growth of *P. gingivalis* at all tested concentrations (Supplementary Figure S2, *p* > 0.05). In contrast, in the *P. gingivalis*–*V. parvula* co-culture system, exogenous nitrate at concentrations of 200 μM and above significantly accelerated the growth of *P. gingivalis* and advanced its entry into the logarithmic growth phase (Figure 5I, *p* < 0.05). qRT-PCR analysis showed that nitrate supplementation significantly upregulated the expression of the gingipain-encoding virulence genes *kgp*, *rgpA*, and *rgpB* in *P. gingivalis* during the late logarithmic growth phase. Among these, *kgp* expression was continuously upregulated with increasing nitrate concentrations, while *rgpA* and *rgpB* expression peaked at 200 μM nitrate (Figure 5J, *p* < 0.05). These results confirm that the epithelial iNOS-derived high nitrate microenvironment enhances the growth and virulence of *P. gingivalis* in a *V. parvula*-dependent manner.

## 3. Discussion

Periodontitis is an inflammatory disease triggered by dysbiotic subgingival biofilms [7]. Factors like smoking and diabetes worsen oral inflammation and disrupt the oral microbiota [26]. Diabetes affects oral health by impairing the host’s barrier function and altering the microbiota’s composition and virulence [27,28,29]. Diabetic periodontitis is linked to a more pathogenic subgingival microbiota, though the exact mechanisms are unclear [30]. Clinical treatments that reduce inflammation may also impact microbiota balance, highlighting a complex relationship between host inflammation and microbial communities [31,32].

Recent research highlights a complex interaction between host inflammation and microorganisms in diseases like ulcerative colitis [33,34]. Periodontitis and diabetes have a mutually worsening relationship, with diabetes-associated periodontitis (DP) patients showing recurrent inflammation and worsening dysbiosis of microbiota compared to those without systemic health issues [35,36]. Using diabetes-related periodontitis as an aggravated inflammatory model, an increase in *P. gingivalis* and *V. parvula* was noted in the subgingival microbiota of DP patients.

Initially, we evaluated the direct regulatory impact of elevated glucose levels on two core bacterial species. *Porphyromonas gingivalis*, a well-documented non-saccharolytic bacterium, is incapable of utilizing carbohydrates and relies entirely on gingipain-mediated protein degradation to acquire amino acids as sources of carbon and nitrogen [37]. In contrast, for *Veillonella parvula*, aside from a limited number of species, the majority of strains are unable to utilize conventional nutrients such as glucose and amino acids, with lactate serving as their primary carbon source [38,39]. These intrinsic metabolic characteristics suggest that hyperglycemia is unlikely to directly influence the growth and virulence of these two bacterial species. As a keystone pathogen in periodontitis, *P. gingivalis* colonizes during the late stages of biofilm formation and is heavily reliant on the support of the bridging microorganism *V. parvula* [40]. Experimental animal studies have demonstrated that, in the absence of *V. parvula*, the capacity of *P. gingivalis* to induce mucosal inflammation is significantly reduced compared to co-inoculation with both bacteria. This finding suggests that *V. parvula* may play a crucial role in mediating enhanced mucosal inflammation in DP.

In subsequent single-cell sequencing analyses, we observed that genes differentially expressed in the DP group, as compared to the P group, were predominantly enriched within epithelial cell subsets. Notably, the *NOS2* gene was primarily expressed in epithelial cells, accompanied by the enrichment of numerous inflammation-related pathways. Building on this observation, we assessed the expression of inducible nitric oxide synthase (iNOS) in its principal expressing cells (epithelial cells and macrophages) in DP and P mice. Our findings confirmed that the upregulation of inflammatory iNOS in the DP group is largely attributable to the increased number of mucosal epithelial cells, which is basically consistent with multiple previous studies [41,42]. Mucosal epithelial cells, serving as the initial barrier to infection, not only regulate local immune responses through mechanisms such as chemokine release and antigen presentation, but also actively participate in shaping of and interaction with the oral microbiota [2,43,44,45]. Furthermore, the close proximity between mucosal epithelial cells and oral microorganisms facilitates metabolic interactions between the two entities.

Inducible nitric oxide synthase (iNOS) has long been regarded as a double-edged sword in mucosal immunity; however, its precise role in host–microbe interactions, particularly within the framework of chronic metabolic inflammation, remains inadequately understood [46]. Under physiological conditions, the expression of iNOS is minimal, and its catalytic product, nitric oxide (NO), serves as a central effector molecule in innate antimicrobial defense, playing a crucial role in protecting the host against invading pathogens [18]. During periods of intense inflammation, elevated expression and activation of cellular iNOS, in conjunction with the redox environment (including superoxide anion and reactive oxygen species) present in local tissues, may create conditions conducive to nitrate biotransformation [47,48]. Furthermore, alterations in the composition of nitrate-reducing microorganisms within the subgingival microbiota of patients with periodontal disease suggest that nitrate may act as a potential mediator through which mucosal epithelial inflammatory iNOS influences oral microbial communities [49,50]. By constructing an in vitro inflammatory gingival epithelial model and analyzing clinical gingival tissue from DP patients, we confirmed that high glucose can lead to iNOS activation in gingival epithelial cells, accompanied by increased secreted nitrate. Adding physiological concentrations of nitrate to the *P. gingivalis–V. parvula* co-culture model enhanced *P. gingivalis* growth and increased the expression of its virulence factors. In contrast, nitrate supplementation did not promote *P. gingivalis* growth in monoculture, indicating that these effects are dependent on the presence of *V. parvula*. Notably, nitrate did not directly promote *V. parvula* proliferation under our experimental conditions. This suggests that the gingival epithelial inflammatory iNOS–nitrate axis in diabetic individuals may be an important mediator by which the host regulates the pathogenic potential of oral microorganisms in the microenvironment. In contrast, the iNOS antagonist molecule Arg-1 exhibits minimal expression within epithelial cell subpopulations. In our study, we were unable to obtain conclusive evidence supporting its central role, indicating that further research is necessary to elucidate its function in epithelial cells.

Recent studies in the field of intestinal diseases have elucidated that inflammation-induced activation of host iNOS and subsequent nitrate production are fundamental mechanisms underlying dysbiosis of the intestinal microbiota. This dysbiosis facilitates the proliferation of *Veillonella* spp. and *Enterobacteriaceae* through pathways reliant on nitrate respiration conversion and specific metabolic profiles [10,20]. Our study confirmed in diabetic individuals that gingival epithelial inflammation reshapes microbial pathogenic potential through nitrate metabolism, suggesting that the iNOS–nitrate axis may be a common therapeutic target for various chronic mucosal inflammatory diseases.

Studies have shown that iNOS-specific inhibitors such as 1400 W have completed preclinical validation in various inflammatory disease models [51,52]. Our study provides a theoretical basis for the application of iNOS inhibitors in the adjuvant treatment of DP. In the future, the expression level of gingival epithelial iNOS, gingival crevicular fluid nitrate concentration, and *V. parvula* abundance in the oral microbiota can be explored as potential biomarkers for the occurrence and development of DP. However, this study still has some limitations. Though clinical baseline characteristics (including disease severity and PLI) were balanced at enrollment, the sample size of the metagenomic sequencing in this study was relatively small. More large-scale observational studies are required to determine the specific changes in the subgingival microbiome of DP patients. Due to conditional constraints, we did not construct epithelial cell-specific *NOS2* conditional knockout mice for verification, and thus cannot completely exclude the interference of iNOS from other cellular sources (such as immune cells) in vivo. To avoid direct mechanical stimulation of periodontal tissues by ligation, the ligature-induced periodontitis model was not adopted, so bacterial colonization was not directly quantified or characterized [53]. However, no bone resorption was observed upon inoculation with the Gram-negative bacterium *V. parvula* alone or in germ-free mice. This suggests that the bone resorption we observed may largely be due to colonization with *P. gingivalis*. In addition, the specific mechanisms and modes of interspecies interaction between *P. gingivalis* and *V. parvula* remain to be clarified. Furthermore, studies have shown that ectopic colonization with *V. parvula* in the intestine can promote intestinal inflammation, suggesting that *V. parvula*, as an opportunistic pathogenic microorganism, exhibits high plasticity in different microenvironments and may play important roles in various mucosal inflammatory diseases [10], which deserves further research and exploration.

## 4. Materials and Methods

### 4.1. Clinical Sample Collection, Metagenomic Sequencing, and Single-Cell RNA Sequencing Analysis

This study was approved by the Ethics Committee of the Hospital of Stomatology, Sun Yat-sen University (Approval No. KQEC-2022-123-01) and was conducted in strict accordance with the principles of the Declaration of Helsinki. All enrolled patients provided written informed consent prior to participation.

A total of 17 subjects were enrolled in this study, including 8 patients with DP (DP group) and 9 patients with non-diabetic chronic periodontitis (P group). Type 2 diabetes was diagnosed according to the 2021 American Diabetes Association (ADA) criteria, with fasting plasma glucose (FPG) > 7.0 mM and surgically acceptable glycemic control defined as FPG ≤ 8.8 mM [54]. The periodontitis classification required Stage III/IV classification per the 2018 AAP criteria [55]. The two groups were matched in terms of age, gender, calculus index (CI), plaque index (PLI), deepest periodontal probing depth, bleeding on probing, and periodontitis staging and grading. Exclusion criteria included patients under 18 or over 70, systemic diseases other than diabetes, dental/oral pathologies (developmental disorders or conditions like Sjögren’s syndrome), recent medication intake, factors influencing the oral microbiota (smoking, pregnancy, lactation, or menstruation), and individuals participating in other clinical trials.

Blood samples from enrolled patients were collected by nurses for glucose testing. A week after scaling, specialists conducted a full-mouth periodontal exam, and an independent clinician recorded periodontal parameters and glucose levels. The participants were categorized into the diabetes-associated periodontitis (DP) and systemically healthy periodontitis (P) groups. Subgingival plaque from Ramfjord index teeth was collected from the deepest pocket sites (>3 mm) using Gracey mini five curettes and stored in liquid nitrogen immediately for subsequent metagenomic sequencing. Gingival tissues obtained from surgical trimming (buccal keratinized gingiva) were preserved in storage medium at 4 °C.

Metagenomic sequencing of 17 subgingival plaque samples (n_DP_ = 8, n_P_ = 9) was performed at BGI, Shenzhen following a standard procedure. DNA samples, concentrated to ≥12.5 ug/uL, were used to construct libraries with 1 μg of DNA. Qualified libraries were sequenced on the BGI DNBSEQ platform. Contigs were assembled with MEGAHIT, and gene sequences within contigs were predicted using MetaGeneMark. The subsequent analysis utilized the Dr. Tom online platform (BGI, China).

Single-cell RNA sequencing was conducted on one sample from each group (DP and P) using the DNBelab C4 platform, which utilizes dual-sized magnetic beads based on Drop-seq. Cell suspensions with over 80% viability were processed with the DNBelab C Series Kit V3.0. Samples, oil, and beads were loaded into C4 chips to generate droplets. mRNA–bead complexes were released via vacuum disruption, and mRNAs were reverse-transcribed into cDNAs. DNA nanoballs (DNBs) were produced through rolling circle amplification. Sequencing was performed using high intensity nanoarrays and cPAS techniques. RNA reads were aligned to a reference genome using STAR software, https://github.com/alexdobin/STAR, accessed on 2 September 2026. Quality control included gene detection and mitochondrial read percentages. Highly variable cell subsets were identified for further analysis. UMAP was used for cluster visualization, and marker genes were identified with the Seurat package’s FindAllMarkers function (logfc.threshold > 0.25, minPct > 0.1, and Padj ≤ 0.05). Clusters were reclassified into known cell types using SCSA, and differential gene expressions were identified with default settings.

### 4.2. Animal Assay

The animal use protocol was reviewed and approved by the Institutional Animal Care and Use Committee (IACUC), Sun Yat-Sen University (Approval No. SYSU-IACUC-2024-B1154). For the diabetes mouse model, 12-week-old males were injected with 50 mg/kg STZ daily for 5 days to induce diabetes, confirmed by blood glucose levels over 200 mg/dL. Controls received sodium citrate buffer. Post-antibiotic clearance, both diabetic and control mice were divided into three groups for oral inoculation with 10^7^ CFU *P. gingivalis*, 10^8^ CFU *V. parvula*, or both every other day for 2 weeks. Repeated bacterial inoculation was performed in the maxillary molar region, with the bacterial suspension applied to the maxillary molars and adjacent gingival tissues. Six weeks later, mice were euthanized for tissue and bone analysis. The maxilla was selected for bone resorption analysis. The maxillary gingiva was harvested for flow cytometry. Experimental details are shown in Figure 2A.

### 4.3. Flow Cytometry of Gingival Tissue

Mouse maxillary gingival tissues were dissected and placed in an enzymatic digestion cocktail (0.15 mg/mL DNase I, 3.2 mg/mL collagenase IV, 2.65 mg/mL dispase II in RPMI 1640 buffer supplemented with 0.8% FBS). The tissues underwent mechanical mincing and digestion at 37 °C for 50 min with 150 rpm agitation. The cell suspension was filtered through a 70 μm strainer, neutralized with FACS buffer, and centrifuged (1200 rpm, 5 min, 4 °C) to obtain single-cell suspensions.

The procedure involved surface staining with BV421-F4/80, AF700-CD45, and BV605-EpCAM antibodies (1 μg/10^6^ cells) and Zombie UV™ dye for 40 min at 4 °C in the dark, followed by washing with FACS buffer. Cells were fixed overnight at 4 °C, washed with permeabilization buffer, and resuspended in 150 μL FACS buffer. Intracellular staining with an APC anti-mouse iNOS antibody was performed for 40 min at 4 °C in the dark. After washing, cells were resuspended in 400 μL FACS buffer for flow cytometry using BD LSRFortessa, and ≥200,000 viable events were analyzed per sample. Gating strategies are shown in Supplementary Figure S1, with data as percentages of positive cells.

### 4.4. Micro-CT Scanning of Mouse Maxillae

According to murine oral inoculation models established previously [56], alveolar bone loss was evaluated exclusively in the maxilla, which served as the standardized anatomical site for morphometric analysis. Maxillae were fixed in 4% paraformaldehyde for 24 h and scanned with a Scano μCT50 system (Scanco Medical AG, Zurich, Switzerland). The micro-CT parameters were as follows: voltage, 70 kV; electric current, 114 μA; and resolution of 10 μm per pixel. Using the manufacturer’s software, three-dimensional reconstructions were analyzed with standardized anatomical orientation to assess bone resorption. Distances from the cementoenamel junction (CEJ) of the first molar (distal) and second molar (mesial) to the alveolar bone crest (ABC) in the interdental space were measured at six sites (bilateral maxillae). The average of these six measurements per animal was calculated as the alveolar bone resorption index.

### 4.5. Immunohistochemical Analysis of Clinical Specimens

Gingiva samples from patients were processed and sectioned at 4 μm. Antigen retrieval was performed with pepsin, and the samples were blocked using 3% H_2_O_2_. Non-specific binding was prevented with 10% normal goat serum. Sections were incubated with IL-1β and TNFα antibodies overnight at 4 °C. Detection was performed using a DAB kit, and slides were scanned at 40× magnification using a Leica Aperio AT2 Scanscope (Leica Biosystems, Vista, CA, USA). Random high power fields were analyzed with ImageJ, Java version 1.8.0_112 (NIH, Bethesda, MD, USA) to count DAB-positive cells against total nucleated cells.

### 4.6. Cell Culture and Stimulation

Human gingival epithelial cells (HGECs) from ATCC were cultured in DMEM/F12 with 10% FBS and penicillin–streptomycin at 37 °C and 5% CO_2_. They were exposed to high glucose DMEM (20 mM) for 6, 12, 24, and 48 h, while the control group received 5.5 mM glucose DMEM. The iNOS inhibitor 1400 W was added to high glucose DMEM at 0.1, 1.0, and 10 μM concentrations. Inhibitor-treated groups received 1400 W in high glucose medium, while the HG control group received only high glucose medium. All treatments were conducted 24 h before performing western blotting, RT-PCR, and nitrate concentration measurements.

### 4.7. RT-PCR Analysis

qRT-PCR followed established protocols [57]. Total RNA was extracted using Nucleozol Reagent (Gene Company Limited, Hong Kong) and reverse transcribed to cDNA with PrimeScript RT Master Mix (Toyobo Co, Ltd., Ōsaka, Japan). PCR was performed on a Bio-Rad CFX96™ Detection System (Roche, Solna, Sweden) with SYBR PCR Master Mix (Roche, Pleasanton, CA, USA). Relative gene expression was quantified using a standard curve, with ACTB as the reference gene. Primer details are shown in Supplementary Table S1.

### 4.8. Western Blotting

Western blotting followed established protocols [57]. Proteins were extracted with RIPA buffer on ice for 30 min and centrifuged for 15 min. Protein concentrations were determined using a BCA assay kit. Proteins underwent SDS-PAGE and were transferred to PVDF membranes, which were blocked with 5% BSA for 1 hour. Primary antibodies were applied, followed by a secondary antibody (1:1000, Abcam, Cambridge, UK), and visualized with a chemiluminescence kit. The antibodies used are detailed in Supplementary Table S2.

### 4.9. Nitrate Concentration Measurement in Culture Supernatant

Nitrate levels in culture supernatants were measured using a CheKine™ Micro Nitric Oxide (NO) Assay Kit (Abbkine, Wuhan, China). After hyperglycemic stimulation of HGECs, supernatants were collected at set intervals. Griess reagent was prepared as per instructions, and a standard curve for nitrate/nitrite was created. For processing, 20 μL of zinc sulfate was added to each sample, the mixture was centrifuged at 14,000 rpm for 10 min at 4 °C, then 100 μL of the supernatant was mixed with Griess reagent and incubated at 37 °C for 30 min. Absorbance was read at 540 nm, and nitrate concentrations were determined from the standard curve.

### 4.10. Bacterial Culture and Co-Culture Experiments

*Porphyromonas gingivalis* W50 and *Veillonella parvula* NCTC1181 were obtained from ATCC, Manassas, VA, USA. *P. gingivalis* was cultured in Brain Heart Infusion (BHI) broth with 1 μg/mL vitamin K1 and 5 mg/L hemin under anaerobic conditions (85% N_2_, 10% H_2_, 5% CO_2_) at 37 °C. *V. parvula* was grown in BHI broth with 2% sodium lactate under equivalent anaerobic conditions. In the co-culture system, an adapted mucin–serum culture medium was used as per Hoare et al. [40] to establish the *V. parvula*–*P. gingivalis* co-culture model, avoiding interference from endogenous heme production by *V. parvula* through the omission of hemin.

### 4.11. Bacterial Genomic DNA Extraction and Absolute Quantification

Total genomic DNA was extracted using a TIANamp Bacteria DNA Kit (DP302, TIANGEN, Beijing, China), with bacterial quantification achieved via 16S rRNA gene-targeted PCR and a Ct-CFU standard curve. Absolute bacterial counts were determined using logarithmic regression analysis.

### 4.12. Statistical Analysis

Experiments were conducted with at least three biological replicates, and data are shown as mean ± SEM. One-way ANOVA with Bonferroni correction was used for single-factor comparisons, while two-way ANOVA with BKY post hoc was used for dual factor comparisons. Analyses were performed and graphs were generated using GraphPad Prism 8.3.0, with significance at *p* < 0.05 (false discovery rate) or *q* < 0.05 (indicated as * for *p* < 0.05).

## 5. Conclusions

Diabetes triggers excessive iNOS-related inflammation and nitrate production in gingival epithelial cells. Increasing nitrate concentration alters the pathogenic potential of oral microbiota in diabetes and contributes to greater *P. gingivalis* virulence and periodontal damage via *V. parvula*. The epithelial iNOS–nitrate pathway is a potential treatment target for diabetes-related periodontitis and inflammatory diseases.

## Figures and Tables

**Table 1:** Clinical baseline characteristics of study subjects.

Characteristic		P	DP	*p* Value
n		9	8	-
Gender (male/female)		5/4	3/5	0.606 ^a^
Age		46.89 ± 9.24	58.00 ± 7.33	0.477 ^b^
CI		2.89 ± 0.33	2.75 ± 0.46	0.159 ^b^
PLI		2.67 ± 0.50	2.25 ± 0.46	0.485 ^b^
Deepest periodontal pocket (mm)		8.56 ± 3.78	8.63 ± 3.11	0.255 ^b^
Bleeding on probing (%)		78.33 ± 16.39	82.50 ± 14.88	0.312 ^b^
Fasting glucose (mM)		-	8.76 ± 2.35	-
Staging and grading of periodontitis	II, B, Generalized	4/9	3/8	0.815 ^b^
	III, C, Generalized	4/9	4/8	
	IV, C, Generalized	1/9	1/8	

^a^, Mann–Whitney U test; ^b^, Student’s *t* test.

## Data Availability

The original contributions presented in this study are included in the article/Supplementary Materials. Further inquiries can be directed to the corresponding authors.

## Supplementary Materials

The following supporting information can be downloaded at: https://www.mdpi.com/article/10.3390/ijms27177934/s1.

## Abbreviations

The following abbreviations are used in this manuscript:

DP	Diabetes-associated periodontitis
*P. gingivalis*	*Porphyromonas gingivalis*
*V. parvula*	*Veillonella parvula*
HGECs	Human gingival epithelial cells
IBD	Inflammatory bowel disease
iNOS	Inducible nitric oxide synthase
NO	Nitric oxide
3-NT	3-Nitro-L-tyrosine
FPG	Fasting plasma glucose
BHI	Brain Heart Infusion
